# Tumor-infiltrating lymphocytes and tumor-associated macrophages as potential predictors of lymph node metastases in major salivary gland cancers

**DOI:** 10.3389/fmed.2023.1163565

**Published:** 2023-07-03

**Authors:** Armando De Virgilio, Maria Vittoria Veneroni, Andrea Costantino, Bianca Maria Festa, Barbara Fiamengo, Daniela Sebastiani, Giuseppe Spriano, Luca Di Tommaso

**Affiliations:** ^1^Department of Biomedical Sciences, Humanitas University, Pieve Emanuele, MI, Italy; ^2^Otorhinolaryngology Unit, IRCCS Humanitas Research Hospital, Rozzano, MI, Italy; ^3^IRCCS Humanitas Research Hospital, Rozzano, MI, Italy; ^4^Pathology Unit, IRCCS Humanitas Research Hospital, Rozzano, MI, Italy

**Keywords:** tumor microenvironment, immunogenicity, immune system, head and neck cancer, immunohistochemistry

## Abstract

**Purpose:**

The purpose of this study is to define if tumor-infiltrating lymphocytes (TILs) and tumor-associated macrophages (TAMs) could represent potential predictors of lymph node metastases (LNM) in salivary gland cancers (SGC).

**Methods:**

A selected number of immunohistochemical markers related to TILs (CD3, CD4, CD68, and FOXP3) and TAMs (CD68 and CD163) were investigated on major salivary gland cancers. TIL and TAM densities were measured on digital images using the open-source QuPath both in the tumor interior (TI) and invasive margin (IM). Correlation with pathologic N classification and follow-up clinical data was investigated.

**Results:**

A total of 25 consecutive patients (men: 11; median age: 62.0) were included. Densities of CD3+ IM (OR = 7.7, 95% CI 1.2–51.2), CD8+ TI (OR = 7.7, 95% CI 1.2–51.2), CD8+ IM (OR = 7.7, 95% CI 1.2–51.2), FOXP3+ TI (OR = 24.0, 95% CI 2.2–255.9), CD68+ TI (OR = 7.7, 95% CI 1.2–51.2), and CD163+ IM (OR = 7.7, 95% CI 1.2 – 51.2), and the Immunoscore CD8/CD3 (OR = 1.9, 95% CI 1.1–3.4) were significantly associated with LNM (*p* < 0.05). CD3+ TI density was significantly associated with tumor recurrence and death (HR = 5.8, 95% CI 1.5–22.6; *p* < 0.05).

**Conclusion:**

A high density of specific TIL and TAM subpopulations might be correlated with a higher probability of LNM in SGC.

## Introduction

Salivary gland cancers (SGCs) are considered rare entities accounting for ~3% of all head and neck malignancies and ~0.3% of all malignant neoplasms (1–3). SGCs collectively represent a very heterogeneous group of neoplasms with complex clinicopathologic characteristics and distinct biological behavior, as demonstrated by the last WHO classification (4).

Lymph node metastases (LNM) represent an important predictor of disease recurrence and survival in SGC (5–7). Current literature shows that the LNM rate depends mostly on tumor stage and grade in addition to the specific histology (8, 9). Therapeutic neck dissection remains an integral part of the neck management protocol in case of clinically or radiologically positive cervical nodes. On the other hand, indications for elective treatment of the neck in clinically node-negative patients remain a controversial topic (10–12). In this context, the possibility to predict the presence of LNM could better define SGC prognosis and the correct management in a more personalized approach.

Recent literature has alluded to the importance of the host immune system in controlling or enhancing tumor progression (13–15). Tumor microenvironment (TME) has shown a broad spectrum of molecular alterations and immunogenic features, which have been recently proposed as potential predictors of patients' prognosis in head and neck cancer (16, 17).

In particular, different tumor-infiltrating lymphocyte (TIL) subpopulations may have a significant role as survival predictors. TILs may be strongly conditioned by their spatial arrangement within the TME and by the expression of inhibitory receptors, which may determine their role in antitumoral or protumoral response (18, 19). On the other hand, tumor-associated macrophages (TAMs) are the major components of innate immune cells that enter tumor tissue, and they may also importantly influence tumor growth and progression in opposite directions, depending on their activation state. TAM may be involved in SGC progression due to their ability to increase tumor angiogenesis, promoting tumor growth and migration (20–22). Many histological varieties and their distinct features have been proven to greatly influence the interaction between cancer and the immune system (Table 1). Indeed, high or low immunogenicity itself cannot be correlated straight away with a specific prognostic value (23).

**Table 1:** Histotypes and their specific tumor immune microenvironment based on the literature analysis.

**Histotype**	**Immunogenicity (23)**
SDC	Immuno-rich TME: higher levels of immune infiltrations compared to other histologies
AdCC	Immuno-poor TME: recurrent and metastatic AdCCs show lower immune infiltration with respect to non-recurrent and non-metastatic AdCCs
MECA	Heterogeneous TME
AciCC	Immune TME enrichment depends on grade: the higher the grade the higher the immunogenicity
AdenoCA	Immuno-rich TME: (mostly CD3+)
ANOS	
MEC	Indifferent immune TME: similar to healthy oral mucosa

SDC, salivary duct carcinoma; AdCC, adenoid-cystic carcinoma; MECA, myoepithelial carcinoma; AciCC, acinic cell carcinoma; AdenoCA, adenocarcinoma; ANOS, adenocarcinoma not otherwise specified; MEC, mucoepidermoid carcinoma.

At this time, there are discordant data regarding the prognostic role of TILs and TAMs in SGCs (23–33). Moreover, there is no study focused on the possibility to predict LNM through TIL and TAM assessments. The purpose of the present study was indeed to perform a preliminary analysis of TIL and TAM to define which markers could represent potential predictors of LNM in SGCs.

## Materials and methods

### Study design

A single-center retrospective study was carried out at the Department of Biomedical Sciences, Otolaryngology—Head & Neck Surgery Unit, Humanitas University, after approval by the institutional review board (Prot. Ne. CE Humanitas 31/22 05/2022). The study was carried out in accordance with the principles of the Declaration of Helsinki, and informed consent was obtained from all the patients.

### Study population

All consecutive patients who underwent surgical treatment for a major salivary gland cancer between January 2009 and December 2020 were included. The inclusion criteria were as follows: (1) age 18 years or older; (2) willing to provide informed consent; (3) diagnosis of major salivary gland cancer; and (4) primary surgical treatment with elective or therapeutic neck dissection. The exclusion criteria were as follows: (1) defects in slides and blocks, (2) insufficient material for pathological analysis, (3) malignant tumors originating from minor salivary glands, (4) diagnosis of squamous cell carcinoma, (5) patients without clinical follow-up or incomplete records, (6) previous treatments (e.g., radiotherapy), (7) tumor recurrences, and (8) distant metastases.

All tumors were staged according to the 8th edition of the American Joint Committee on Cancer (AJCC) staging manual (34). In the case of patients treated before 2017, the histological reports were reviewed to adequately re-stage the tumors. The decision to perform adjuvant treatments was made according to the National Comprehensive Cancer Network (NCCN) guidelines (35). The clinical charts of these patients were retrospectively reviewed to collect the following clinical data: demographic characteristics (age and sex); tumor site and grade; clinical and pathological staging; lymph node yield and ratio (LNY and LNR); adjuvant treatments; and follow-up data.

### Histopathological analysis

Histological slides obtained were retrieved from a pathological archive. All of the H&E slides were re-examined by an experienced pathologist (L.D.T.) in order to choose the most representative tumor sample which had to contain both tumor and peritumoral salivary gland tissues. The corresponding formalin-fixed, paraffin-embedded tissue blocks were retrieved. Consecutive 4 μm slices were used to create slides for IHC staining.

### Immunohistochemical analysis

**Figure 1:**
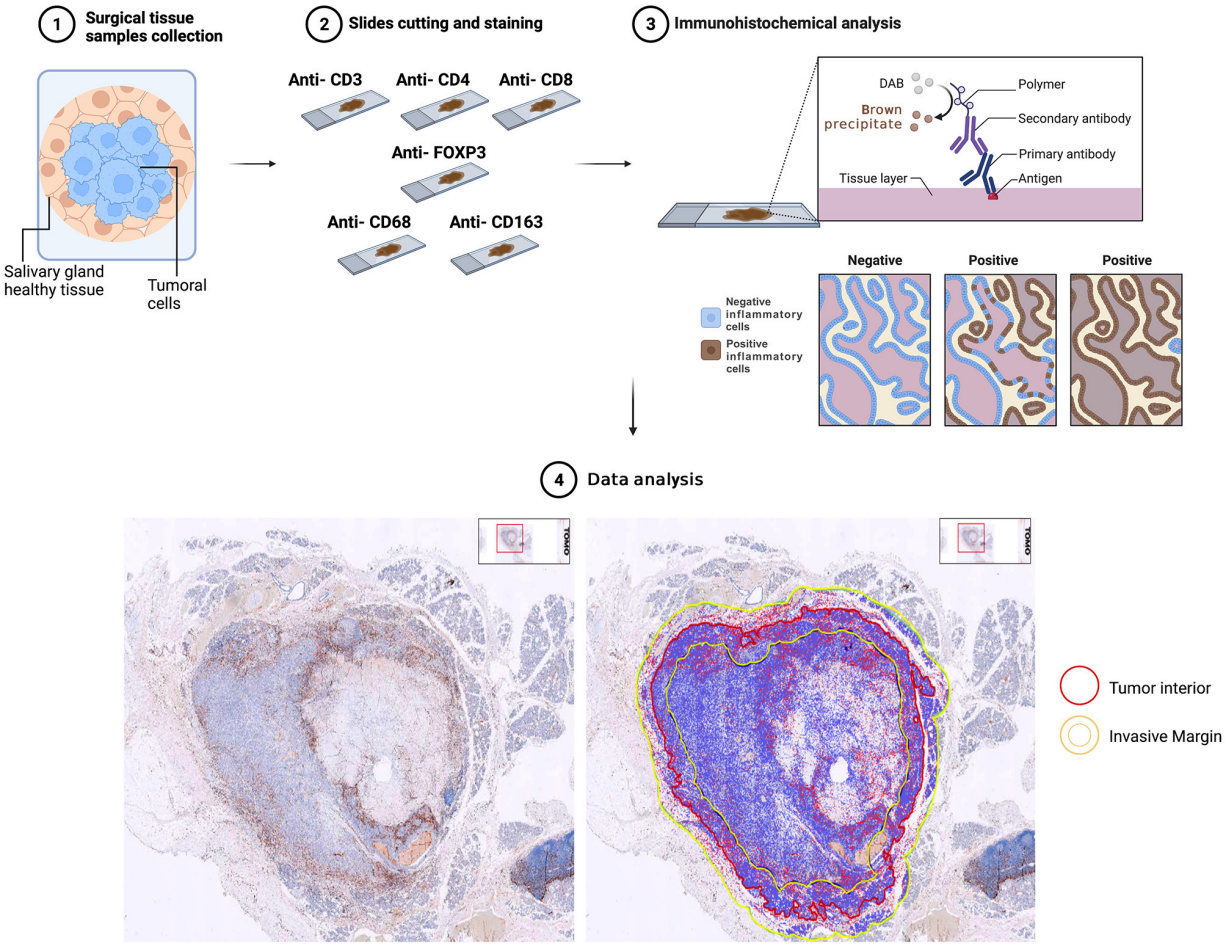
Flowchart of the immunohistochemical analysis performed on surgical tissue samples.

Slides were stained using the BOND-III Staining System (Leica Biosystems) along with anti-CD3 (LN10, Leica Biosystems), anti-CD4 (4B12, Leica Biosystems), anti-CD8 (4B11, Leica Biosystems), anti-CD68 (514H12, Leica Biosystems), anti-CD163 (10D6, Leica Biosystems), and anti-FOXP3 (236A/E7, Leica Biosystems) monoclonal antibodies and the BOND Polymer Refine Detection (Leica Biosystems), following the manufacturer's instruction. After immunohistochemical staining, the slides were scanned using the Philips IntelliSite UltraFast Scanner (Philips Digital Pathology Solutions, Best, the Netherlands). Digitalized slides were then imported into the open-source program QuPath (ver. 0.3.2) for further image analysis (36). The field of interest was divided into two different regions represented by the tumor interior (TI), and the tumor invasive margin (IM) defined as 500 μm outward and 500 μm inward of the tumor border. Each tumor area was computed using QuPath, which detects single cells by using a built-in cell segmentation algorithm. Finally, the numbers of tumor cells and positive stained immune cells were automatically counted within each region (Figure 1).

### Quantification of inflammatory cells

The number of positively stained cells per tissue surface unit in mm (2) was separately measured in TI and MI. The density of positive lymphocytes or macrophages was defined as the number of positively stained lymphocytes or macrophages per mm (2).

The densities of each single marker and different ratios between markers of the same family were used as summary measures. In particular, macrophages were measured as the density of CD163 in TI and IM and the density of CD68 in TI and MI, while the ratios were calculated between CD163/CD68 densities in TI and MI. The same approach was used with lymphocytes, among which the ratio of densities was as follows: CD8/CD3, CD4/CD3, and FOXP3/CD3.

**Figure 2:**
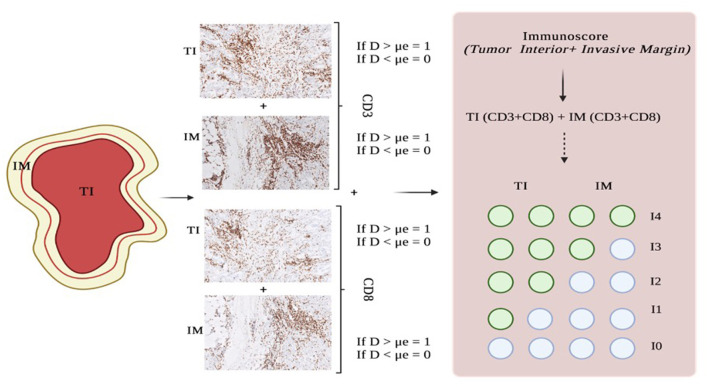
Steps for measuring the Immunoscore. Histopathological slides show CD3 and CD8 immunostaining on an intermediate-grade SDC from our cohort. IM, invasive margin; TI, tumor interior; D, density; μe, median.

To stratify patients into groups based on the degree of tumor lymphocyte infiltration, the median of each TIL density was used to define the “Immunoscore”, as already proposed for other malignant tumors (37). Based on the established threshold, each patient was given a binary score (0 if ≤ μe, 1 if >μe) for each immune cell type (CD3+, CD8+, and CD4+), in each tumor region (TI and IM). In particular, two different Immunoscores were measured for both CD8-CD3 and CD4-CD3 correlations. The Immunoscore for each patient was calculated by adding the four binary score values on a scale from 0 to 4. Five patient groups were defined as follows: patients with high densities of CD3+ and CD8+ or CD4+ cells in both regions (All-High) were classified as Im4; patients with low density for both markers (All-Low) were classified as Im0; and patients with one (1-High), two (2-High), and three (3-High) among these two markers were classified as Im1, Im2, and Im3, respectively (Figure 2).

### Statistical analysis

Patients' demographics, tumor characteristics, and data derived from the IHC analysis were collected and stored in a Microsoft Excel^®^ spreadsheet.

Categorical variables were summarized by counts and percentages, while continuous variables were reported as means ± standard deviations (SD) or as median ± interquartile range (IQR: 25th and 75th) if the values were not normally distributed at the Shapiro–Wilk normality test (38).

TIL and TAM densities and TIL and TAM ratios were dichotomized according to the median value. In particular, tumors with TIL/TAM densities and TIL/TAM ratios lower or equal to the median value were classified as “low”, and tumors with TIL/TAM densities and TIL/TAM ratios higher than the median were classified as “high”. TIL and TAM densities, TIL and TAM ratios, and Immunoscore values were correlated with the risk of LNM and patients' prognosis.

The primary outcome was represented by the presence of LNM in the final histopathological report. The secondary outcomes were as follows: disease-free survival (DFS), defined as the time from surgery to death or tumor recurrence; overall survival (OS), defined as the time from surgery to death from any cause; and disease-specific survival (DSS), defined as the time from surgery to death from cancer.

Correlation with the pathologic *N* classification was analyzed using univariate binary logistic regression models, and the results were summarized with odds ratios (ORs) and 95% confidence intervals (CIs). DFS, OS, and DSS were estimated using a Kaplan–Meier analysis (39). A univariable analysis was performed using the log-rank test to identify potential prognostic factors of DFS. After testing for the proportional hazard assumption on the basis of the Schoenfeld residuals (40), a univariable analysis was also conducted using the Cox proportional hazards regression model (41), and the results were summarized with hazard ratios (HRs) and 95% confidence intervals (CIs). A multivariable regression model was not applied due to the low sample and low rate of events in all outcomes (42).

Statistical analyses were performed using R software for statistical computing (R version 4.0.1) and GraphPad Prism v 6.0 (GraphPad Software Inc., La Jolla, CA, USA). A *p-*value of < 0.05 was considered to indicate statistical significance.

## Results

### Patients' characteristics

After applying the abovementioned inclusion and exclusion criteria, a total of 25 consecutive patients (men: 11; median age: 62.0, IQR: 49.0–73.5) were included in the final analysis. The majority of patients suffered from parotid cancer (*n* = 20, 80%). The most common histotypes were salivary duct carcinoma (*n* = 7, 28%), adenoid cystic carcinoma (n = 6, 24%), and mucoepidermoid carcinoma (*n* = 4, 16%). A similar proportion of locally early (T1-2; *n* = 12, 48%) and locally advanced (T3-T4; *n* = 13, 52%) tumors was detected. The majority of tumors (*n* = 15, 60%) showed high histological grade. A total of 17 (68%) patients underwent elective neck dissection, while the remaining 8 (32%) patients underwent therapeutic neck dissection. Overall, the median number of lymph nodes retrieved (LNY) was 24.0 (IQR: 5.0–36.5). Nine (36%) patients showed evidence of lymph node metastases at the final histopathological assessment. The median number of positive lymph nodes was 2.0 (IQR: 1.0–3.5), with a median LNR of 0.07 (IQR: 0.03–0.11). Postoperative RT was performed in 19 (76%) patients, while concurrent CRT was administered in 1 (4%) patient.

Demographic data and clinicopathological features of the entire cohort are presented in Table 2.

**Table 2:** Demographic data and clinicopathological features of the entire cohort.

**Clinical feature**	***n* (%) or median (IQR)**
**Gender**	
Male	11 (44%)
Female	14 (56%)
Age at diagnosis	62 (49–73.5)
**Primary tumor site**	
Parotid	20 (80%)
Submandibular gland	4 (16%)
Sublingual gland	1 (4%)
**Tumor histology**	
Mucoepidermoid carcinoma	4 (16%)
Salivary duct carcinoma	7 (28%)
Adenoid cystic carcinoma	6 (24%)
Adenocarcinoma not otherwise specified	2 (8%)
Acinic cell carcinoma	2 (8%)
Myoepithelial carcinoma	1 (4%)
Epithelial-myoepithelial carcinoma	1 (4%)
Carcinoma ex-pleomorphic adenoma	1 (4%)
Poorly differentiated carcinoma	1 (4%)
***T*** **classification**	
T1	2 (8%)
T2	10 (40%)
T3	7 (28%)
T4a	6 (24%)
***N*** **classification**	
N0	16 (64%)
N1	2 (8%)
N2b	6 (24%)
N3b	1 (4%)
***M*** **classification**	
M0	25 (100%)
M1	0
**Grade**	
Low	6 (24%)
Intermediate	4 (16%)
High	15 (60%)
**Treatment**	
Surgery	5 (20%)
Surgery + RT	19 (76%)
Surgery + CRT	1 (4%)
Clinical follow-up	29 (17.5–58.5)
**Disease status**	
No evidence of disease	17 (68%)
Local recurrence	2 (8%)
Regional recurrence	4 (16%)
Distant recurrence	6 (24%)
Death for other causes	3 (12%)
Death for disease	4 (16%)

Data are presented as counts and percentages or as median ± interquartile range. IQR, interquartile range; RT, radiotherapy; CRT, chemo-radiotherapy.

### Survival analysis

**Figure 3:**
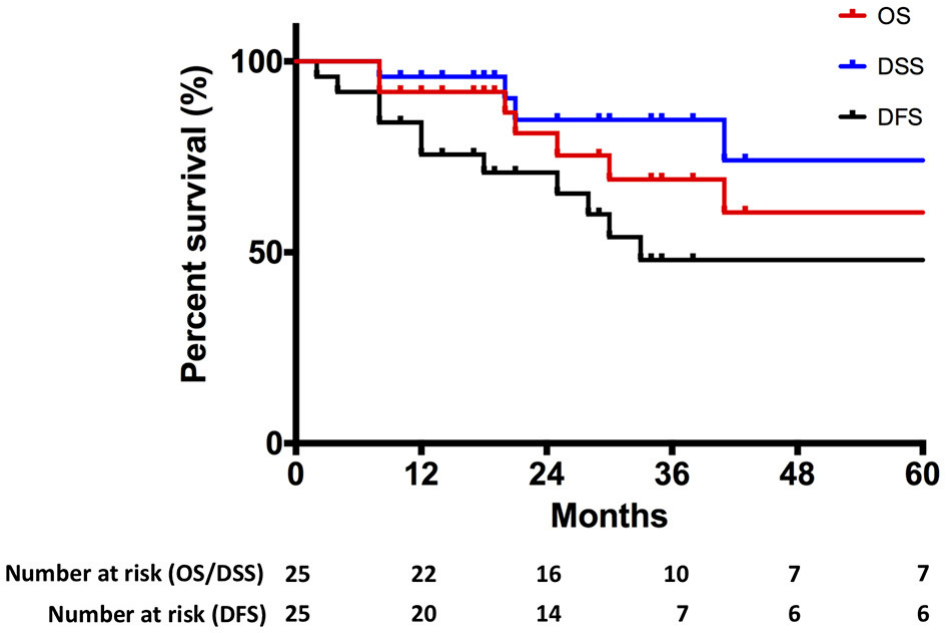
Kaplan–Meier curve representing overall survival (OS), disease-specific survival (DSS), and disease-free survival (DFS) of the entire cohort.

The median follow-up time was 29.0 months (IQR: 17.5–58.5). Seven patients died during follow-up after primary treatment, including four patients who died of cancer. In total, 17 patients showed no evidence of disease at the last follow-up. Estimated DFS rates (95% CI; number still at risk) at 1 and 3 years were 75.6% (53.5–88.2; 20) and 48.0% (25.1–67.7; 7), respectively. Estimated OS rates (95% CI; number still at risk) at 1 and 3 years were 92.0% (71.6–97.9; 22) and 69.1% (42.9–85.1; 10), respectively. Estimated DSS rates (95% CI; number still at risk) at 1 and 3 years were 96.0% (74.8–99.4; 22) and 84.7% (59.2–94.9; 10), respectively. Summary survival curves are presented in Figure 3.

### Immunological markers as predictors of prognosis and lymph node metastases

**Figure 4:**
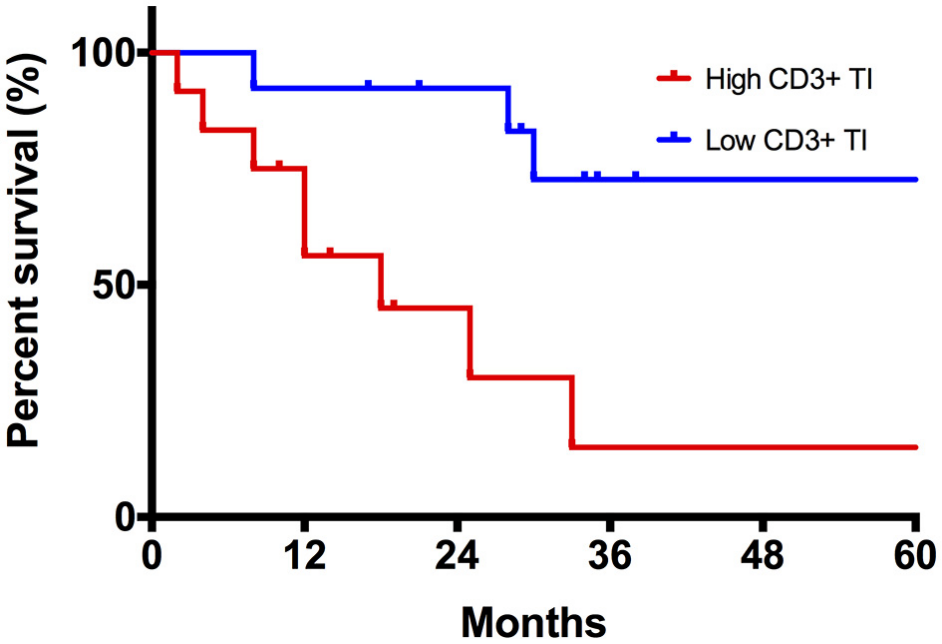
Kaplan–Meier curve comparing disease-specific survival (DFS) between high and low CD3+ TI density tumors.

The results from the univariable Kaplan–Meier and Cox regression analyses for TILs and TAMs are shown in Tables 3, 4, respectively. The only feature with a significant association with DFS was the density of CD3+ at TI. As shown in Figure 4, patients with a high CD3+ density at TI showed a higher risk of death or tumor recurrence compared with patients with low CD3+ density at TI (HR = 5.8, 95% CI 1.5–22.6; *p* < 0.05). The other investigated features did not correlate with death or tumor recurrence.

**Table 3:** Univariable Kaplan–Meier analysis and univariable Cox regression analysis results for TIL data.

			**Kaplan-Meier analysis**			**Cox regression analysis**		
**Feature**		***N***	**1-y DFS (%)**	**3-y DFS (%)**	***p*** **-value**	**HR**	**95% CI**	***p*** **-value**
Density CD3+ TI^*^	Low	13	92.3	72.7	< 0.05	5.8	1.5–22.6	< 0.05
	High	12	56.2	15				
Density CD3+ IM	Low	13	84.6	61.7	0.13	2.5	0.7–8.6	0.14
	High	12	65.6	32.8				
Density CD8+ TI	Low	13	84.6	64.5	0.08	2.8	0.8–9.8	0.10
	High	12	64.8	27				
Density CD8+ IM	Low	13	76.9	56.1	0.42	1.6	0.5–5.3	0.43
	High	12	74.1	38.1				
Density CD4+ TI	Low	13	83.9	46.6	0.69	1.3	0.4–4.2	0.69
	High	12	66.7	47.6				
Density CD4+ IM	Low	13	76.1	33.3	0.31	0.5	0.2–1.8	0.32
	High	12	75	62.5				
Density FOXP3+ TI	Low	13	84.6	63.5	0.13	2.5	0.7–8.8	0.14
	High	12	64.8	28.8				
Density FOXP3+ IM	Low	13	69.2	50.5	0.94	0.9	0.3–3.2	0.94
	High	12	81.8	42.1				
Ratio CD8/CD3 TI	Low	13	69.2	55.4	0.69	1.3	0.4–4.2	0.69
	High	12	83.3	42.3				
Ratio CD8/CD3 IM	Low	13	69.2	50.5	0.89	1.1	0.3–3.6	0.89
	High	12	82.5	42.4				
Ratio CD4/CD3 TI	Low	13	76.1	34.8	0.53	0.7	0.2–2.3	0.53
	High	12	75	56.2				
Ratio CD4/CD3 IM	Low	14	78.6	39.9	0.51	0.7	0.2–2.3	0.52
	High	11	72.7	58.2				
Immunoscore CD8/CD3	0	8	87.5	54.7	0.15	1.4	1.0–2.0	0.07
	1	3	100	100				
	2	5	80	60				
	3	0	-	-				
	4	9	64.8	16.2				
Immunoscore CD4/CD3	0	3	66.7	33.3	0.07	1.5	0.9–2.6	0.13
	1	4	100	100				
	2	10	88.9	51.8				
	3	3	100	50				
	4	5	20	20				
Ratio FOXP3/CD3 TI	Low	13	76.9	48.8	0.92	1.1	0.3–3.5	0.92
	High	12	74.1	46.3				
Ratio FOXP3/CD3 IM	Low	13	76.9	35.2	0.44	0.6	0.2–2.1	0.45
	High	12	73.3	61.1				

DFS, disease-free survival; HR, hazard ratio; CI, confidence interval; TI, tumor interior; IM, invasive margin. The ^*^ symbol indicates the p-value that is statistically significant.

**Table 4:** Univariable Kaplan–Meier analysis and univariable Cox regression analysis results for TAM data.

			**Kaplan-Meier analysis**			**Cox regression analysis**		
**Feature**		***N***	**1-y DFS (%)**	**3-y DFS (%)**	***p*** **-value**	**HR**	**95% CI**	***p*** **-value**
Density CD68+ TI	Low	13	76.9	54.9	0.48	1.5	0.5–5.0	0.48
	High	12	74.1	39.7				
Density CD68+ IM	Low	13	84.6	56.1	0.48	1.5	0.5–5.0	0.48
	High	12	73.3	36.7				
Density CD163+ TI	Low	13	84.6	61.7	0.13	2.5	0.7- 8.6	0.14
	High	12	65.6	32.8				
Density CD163+ IM	Low	13	76.9	52.7	0.57	1.4	0.4–4.6	0.58
	High	12	74.1	42.3				
Ratio CD163/CD68 TI	Low	13	83.9	54.4	0.46	1.6	0.5–5.1	0.46
	High	12	66.7	40				
Ratio CD163/CD68 IM	Low	13	84.6	62.7	0.17	2.3	0.7–7.8	0.19
	High	12	65.6	32.8				

DFS, disease-free survival; HR, hazard ratio; CI, confidence interval; TI, tumor interior; IM, invasive margin.

Table 5 summarizes the correlation between LNM and TIL. In particular, densities of CD3+ IM, CD8+ TI, CD8+ IM, FOXP3+ TI, and the Immunoscore CD8/CD3 were the parameters significantly associated with LNM by univariable analysis. Table 6 summarizes the correlation between LNM and TAM. In particular, CD68+ TI and CD163+ IM showed a significant association with the presence of LNM.

**Table 5:** Results of univariate binary logistic regression models assessing the correlation between pathologic *N* classification and TIL data.

**Feature**	**Median (IQR)**	**OR (95% CI)**	***p* value**
Density CD3+ TI	135.3 (64.9–357.8)	3.3 (0.6–18.5)	0.17
Density CD3+ IM^*^	303.7 (230.4–659.5)	7.7 (1.16–51.2)	< 0.05
Density CD8+ TI^*^	76.8 (53.9–161.5)	7.7 (1.16–51.2)	< 0.05
Density CD8+ IM^*^	147.6 (117.8–319.9)	7.7 (1.16–51.2)	< 0.05
Density CD4+ TI	6.1 (2.2–13.0)	1.6 (0.3–8.3)	0.57
Density CD4+ IM	9.3 (4.1–25.7)	0.8 (0.2–4.1)	0.79
Density FOXP3+ TI^*^	22.4 (8.5–36.9)	24.0 (2.2–255.9)	< 0.05
Density FOXP3+ IM	42.3 (17.4–79.9)	3.3 (0.6–18.5)	0.17
Ratio CD8/CD3 TI	0.58 (0.5–0.8)	0.8 (0.2–4.1)	0.79
Ratio CD8/CD3 IM	0.55 (0.4–0.7)	3.3 (0.6–18.5)	0.17
Ratio CD4/CD3 TI	0.04 (0–0.2)	0.8 (0.2–4.1)	0.79
Ratio CD4/CD3 IM	0.04 (0–0.1)	1.0 (0.2–5.3)	0.97
Ratio FOXP3/CD3 TI	0.15 (0–0.2)	1.6 (0.3–8.3)	0.57
Ratio FOXP3/CD3 IM	0.11 (0–0.2)	0.8 (0.2–4.1)	0.79
Immunoscore CD8/CD3^*^	-	1.9 (1.1–3.4)	< 0.05
Immunoscore CD4/CD3	-	1.6 (0.8–3.2)	0.20

The results are reported as OR and 95% CI. IQR, interquartile range; OR, odds ratios; CI, confidence interval; TI, tumor interior; IM, invasive margin. The ^*^ symbol indicates the p-value that is statistically significant.

**Table 6:** Results of univariate binary logistic regression models assessing the correlation between pathologic N classification and TAM data.

**Feature**	**Median (IQR)**	**OR (95% CI)**	***p*-value**
Density CD68+ TI^*^	57.4 (25.5–180.2)	7.7 (1.16–51.2)	< 0.05
Density CD68+ IM	73.8 (38.2–108.5)	3.3 (0.6–18.5)	0.17
Density CD163+ TI	321.8 (157.2–640.8)	3.3 (0.6–18.5)	0.17
Density CD163+ IM^*^	424.9 (249.2–736.4)	7.7 (1.16–51.2)	< 0.05
Ratio CD163/CD68 TI	4.5 (2.3–8.9)	0.4 (0.1–2.1)	0.28
Ratio CD163/CD68 IM	6.4 (3.5–8.7)	0.8 (0.2–4.1)	0.79

The results are reported as OR and 95% CI. IQR, interquartile range; OR, odds ratios; CI, confidence interval; TI, tumor interior; IM, invasive margin. The ^*^ symbol indicates the p-value that is statistically significant.

## Discussion

To the best of our knowledge, this is the first study that investigates the potential role of TIL and TAM assessments for the prediction of LNM in SGCs. Our analysis showed that a high density of specific TIL and TAM subpopulations is correlated with a higher probability of LNM. In particular, intratumoral CD68+ and marginal CD163+ TAM were associated with lymph node spreading. According to the current literature, M2 (CD163+) polarized TAM has been described as an inducer of tumor migration and invasion in SGCs (24, 43). Poor immunological SGCs (e.g., adenoid cystic carcinoma) have been demonstrated to have a much greater amount of protumoral M2 polarized TAM, with potential implications in terms of disease control. Moreover, higher CD163 expression may also be used to assess the activity of myeloid-derived suppressor cells (MDSCs), resulting in synergic protumoral activity and an increased risk of tumor recurrence (43). On the other hand, a positive correlation between CD68+ TAM and tumoral angiogenic activity was found in SGCs. In particular, a high density of CD68+ TAM was associated with a higher density of micro-vessels and vascular endothelial growth factor A (VEGF-A), markers of vascular neovascularization and tumor remodeling (24). Accordingly, Dutsch-Wicherek et al. found that CD68 positivity was higher in tumors with LNM compared with N0 cases in a cohort of 35 patients with salivary gland adenocarcinoma (44). Hence, CD68+ and CD163+ TAM might collaborate in assisting tumoral cells to leave the primary tumor and invade lymph nodes due to the increasing vascularization and the induction of tumoral cell spreading. Considering the role of TIL, intratumoral and marginal CD8+, intratumoral FOXP3+, and marginal CD3+ showed to be predictive of LNM. Hiss et al. found significant evidence of the increased occurrence of LNM in high-grade acinic cell carcinomas, which also have a greater CD3+ expression with respect to low-grade tumors (30). In line with our findings, Kesar et al. described that tumors with a high total CD8+ showed aggressive behavior in terms of nodal involvement (32). Our results are in line with the concept of CD8+ cell exhaustion due to the continuous exposition to antigen and/or inflammatory signals (45). In addition, Immunoscore for CD8/CD3 was also related to LNM, validating the evidence of an increased risk of tumor progression when total CD3 positively marked cells present an important percentage of CD8+ co-antigen. In particular, this may be a further confirmation of the possible T-cell dysfunction because of inhibitory receptor expression (46, 47).

As stated above, the presence of LNM is an important prognostic predictor in SGCs (5–7). However, we did not find a direct correlation between TIL/TAM and DFS in our cohort. In particular, only a high CD3+ density at TI was associated with a higher risk of death or tumor recurrence. Literature data are heterogeneous in terms of the prognostic role of CD3+ cells, but a high density of CD3+ was found to be associated with better survival in the ovarian (48), endometrial (49), and colon (50) carcinomas. A high value of CD3+ density could be related to an increase in immunostimulatory TME, represented mostly by cytotoxic CD3+CD8+ cells and Th1-polarized CD3+CD4+ cells. On the other hand, high CD3+ density could be associated with an immunosuppressive TME, composed of Treg (CD3+CD4+FOXP3+) and Th2 polarized CD3+CD4+. Indeed, a greater lymphocytic tumor microenvironment is characterized by increased T-cell dysfunction due to increased expression of inhibitory receptors such as LAG3, PDL1, PDL2, TIM3, and CTLA4+ by lymphocytic cells, as demonstrated in a previously published study by Linxweiler et al. (43). The increased density of TILs is followed by an increase in PDL1 expression, suggesting that lymphocyte exhaustion could play a critical role in the cessation of the antitumoral response of inflammatory infiltration. For these reasons, our results are difficult to interpret, and further studies are needed to better define the prognostic role of CD3+ cells in SGCs. According to current literature data, CD8+ cytotoxic cells seem to have the greatest impact on tumor survival among all TILs subpopulations. However, contradictory results are reported by different studies, investigating the prognostic effect of intratumoral\peritumoral CD8+ cell density and location. Mosconi et al. found that a high CD8+ cell density in the invasive front displayed significantly poor OS (33), while Kesar et al. recently demonstrated that the amount of CD8+ cells in the intratumoral compartment displayed an indifferent impact on patient survival (32). Accordingly, other studies found no significant evidence of CD8 + TIL density correlation with patient prognosis (26, 28, 51). The specific role of CD4+ T-cell subsets has been rarely explored in SGCs. A higher CD4+ expression compared with CD8+ TIL (CD4/CD8 ratio up to 10:2) was found to be associated with a better prognosis in the salivary duct carcinoma (52), despite this disproportion having no evidence in other SGC histologies that are usually defined by a general predominancy of CD8+ T cells (53, 54). Immunosuppression and T-cell exhaustion by CD4+ cells have been found to be mainly encouraged by FOXP3 upregulation on SGC specimens and in peripheral blood samples (26, 29). However, no studies demonstrated a correlation between FOXP3+ cells and survival in SGCs. Finally, TAMs have been taken into consideration as a potential tool for prognosis prediction, as already stated. However, only a few studies investigated their prognostic significance in SGCs (24, 43, 44), and further data are needed to clarify their role.

Due to the limited sample size of our study, a definitive correlation between nodal metastasis and TME alone cannot be established, as other known determinants such as histology and grading must also be considered. However, our study yielded promising results suggesting that TME may serve as a valuable adjunctive indicator. In contrast to the literature which depicts MEC as an indifferent SGC in terms of immune infiltration (32), all the four MECs which were enrolled in our study showed a high expression of immune infiltrates, especially CD3+ and CD8+ in both tumoral interior and invasive margin. In addition, three out of four presented locoregional recurrences or distant recurrences and all these showed high anti-CD3+ staining. On the other hand, the only low-grade MEC with no recurrences during the follow-up showed low CD3 positivity. Interestingly, among the seven SDCs analyzed, three out of five high-grade samples did not exhibit lymph nodal involvement and had low levels of total TAMs and TILs, while both intermediate and low-grade samples showed positive lymph nodal metastasis and high levels of TAMs and TILs. These findings are noteworthy as they challenge the conventional belief that tumor grade is a decisive factor in determining outcomes.

The main limitations of this study are represented by the small sample size and the retrospective nature of the analysis. As stated above, various SGC histologies harbor a different rate of metastatic spread, and we were not able to perform multivariable analysis to adjust for this important covariate. Given the rarity and great heterogeneity of SGC, multicentric studies are mandatory to investigate if TIL and TAM are independent predictors of LNM. In our study, we included only patients with tumors arising from major salivary glands to avoid confounding our results based on different regional lymphatic drainage and risk of LNM (e.g., oral cavity tumors). Moreover, we had to exclude an important proportion of patients who did not undergo neck dissection, even if a definitive diagnosis of malignant SGC was found in the final histopathological report. However, this monocentric study was conducted at a feasibility level with a wide assessment of TIL and TAM markers with the primary aim to define their potential role in LNM prediction. In addition, although the retrospective analysis of tissue samples may represent a source of bias, we used a more objective counting tool (QuPath) in order to obtain a more clear and defined methodology and standardization of TIL/TAM quantification. From this perspective, the small sample size did not allow to perform further analysis (e.g., ROC analysis) to define the best cutoff value to distinguish low and high TIL and TAM densities. In fact, further studies should focus on standardizing the definition of low and high density of TIL and TAM, using larger samples.

## Conclusion

TILs and TAMs are potential predictors of LNM in SGCs. In particular, a high density of specific TIL and TAM subpopulations might be correlated to a higher probability of LNM. On the other hand, no clear association was found between TIL/TAM and patients' prognosis in terms of tumor control or survival. Further multicentric prospective studies are mandatory to better define the role of TIL and TAM assessments in the management of SGCs.

## Data availability statement

The raw data supporting the conclusions of this article will be made available by the authors, without undue reservation.

## Ethics statement

A single-center retrospective study was carried out at the Department of Biomedical Sciences, Otolaryngology-Head and Neck Surgery Unit, Humanitas University, after approval by the Institutional Review Board (Prot. Ne. CE Humanitas 31/22 05/2022). The study was carried out in accordance with the principles of the Helsinki Declaration, and informed consent was obtained from all patients.

## Author contributions

AD, AC, GS, and LD: study concept. AD, MV, AC, and LD: study design. MV, BMF, BF, and DS: data acquisition. AC: data analysis. AD, MV, and AC: manuscript preparation. BMF, BF, DS, GS, and LD: manuscript editing. All authors contributed to the article and approved the submitted version.
